# Effects of High-Intensity Training of Professional Runners on Myocardial Hypertrophy and Subclinical Atherosclerosis

**DOI:** 10.1371/journal.pone.0166009

**Published:** 2016-11-11

**Authors:** Célia Regina de Oliveira Bittencourt, Maria Cristina de Oliveira Izar, Valdir Lauro Schwerz, Rui Manuel dos Santos Póvoa, Henrique Andrade Rodrigues Fonseca, Marília Izar Helfenstein Fonseca, Henrique Tria Bianco, Carolina Nunes França, Carlos Eduardo dos Santos Ferreira, Francisco Antonio Helfenstein Fonseca

**Affiliations:** 1 Cardiology Division, Federal University of Sao Paulo, Sao Paulo, Brazil; 2 University of Santo Amaro, Sao Paulo, Brazil; 3 Albert Einstein Israeli Hospital, Sao Paulo, Brazil; Temple University, UNITED STATES

## Abstract

To evaluate the effects of long-term exposure to high-intensity training among professional runners on cardiac hypertrophy and subclinical atherosclerosis.

Prospective study included runners of both sexes (n = 52) and age and gender matched controls (n = 57), without classical cardiovascular risk factors. Ventricular hypertrophy was quantified by echocardiography by linear method and carotid intima-media thickness (cIMT) by 2-D images obtained by ultrasonography. Endothelial function was evaluated by flow-mediated dilation (FMD). Steroid hormones were quantified by HPLC followed by LC-MS/MS. Higher left ventricular (LV) mass index was found in male athletes (p<0.0001 vs. other groups). When adjusted for gender, the degree of left ventricular mass index classified as mildly, moderately or severely abnormal was obtained in 26%, 35%, and 30%, respectively, of female athletes, and in 39%, 14%, and 21%, respectively, of male athletes. Higher ratio of the early (E) to late (A) ventricular filling velocities was found in athletes of both genders. Male athletes presented lower cIMT in the right (p = 0.012 vs. male controls) and left (p<0.0001 vs. male controls) common carotid arteries, without differences in cIMT between female athletes and controls. FMD results were similar among groups. Higher serum testosterone levels were found in male athletes (p<0.0001 vs. other groups) and they were correlated with LV mass (r = 0.50, p<0.0001). The chronic exposure of high-intensity training among professional runners of both genders was associated with increased ventricular mass and adaptive remodeling. Less subclinical atherosclerosis was found in male athletes. Differences in steroid hormones may account in part for these findings.

## Introduction

It is well known that long-term exposure to high-intensity exercise may promote cardiac remodeling, involving all cavities [1,2]. However, some geometric patterns of the so called “athlete’s heart” may differ among individuals submitted to same training and competition level, and according to the modality of high-performance exercise [3,4]. Specifically, endurance athletes commonly exhibit eccentric hypertrophy with balanced increase in chambers and walls, while concentric hypertrophy has been reported mainly for resistance athletes [5,6]. The hypothesis for these differences seems related to differences in the pattern of hemodynamic load between these modalities of exercise [7].

It is controversial whether long-term exposure to intensive training prevents the development of atherosclerosis or promotes healthy vascular remodeling, despite substantial benefits on body mass, body fat and biochemical parameters [8]. In fact, similar degree of subclinical atherosclerosis has been reported between athletes and controls [9,10]. In addition, lower rates of cardiovascular disease have been described in pre-menopausal women, in the general population [11], but female athletes may have hormonal disturbances that can abolish the vascular protection against atherosclerosis [10,12]. Furthermore, flow-mediated dilation, an endothelial vascular marker capable to predict long-term cardiovascular events [13], seems impaired in athletes, possibly due to differences in artery size and wall thickness [14].

Our study addresses two important topics in this field. It examines the effects of chronic intensive training in the left ventricular mass and carotid intima-media thickness, and reports differences by gender between these athletes.

## Materials and Methods

### Study population

Fifty-five professional half-marathon runners and 57 age and gender matched controls, without known cardiovascular disease were consecutively included in the study. These athletes were classified as professional runners because they have been either registered in the athletic federation as elite runners or have received financial support for their full dedication to training and races.

Indeed, subjects with cardiovascular risk factors such as hypertension, diabetes, obesity, smoking, or hypercholesterolemia were excluded. The local ethics committee of the Federal University of Sao Paulo (Brazil) approved the study (# 1808/08) and all participants have signed the written informed consent prior to their inclusion in the study protocol.

### Laboratory assays and dietary intake

Blood samples were collected on Thursdays, before exercise and after 12-hour fasting period and the laboratory analyses were performed at the central laboratory of our university. All these analyses were obtained without interruption of the training program, aiming to mirror the athletes’ real lives. Sex hormones were quantified by high performance liquid chromatography (HPLC) followed by mass spectrometry (LC-MS/MS).

A 24-h recall of dietary intake was obtained to estimate the content and type of macronutrients (fat, protein, carbohydrate, cholesterol, fibers). Information regarding menstrual cycle was obtained for females in the same day of blood collection.

### Ultrasound measurements

For the echocardiographic parameters, the recommendations of the American Society of Echocardiography were used, including the reference limits and partition values for left ventricular mass and geometry by gender, obtained by linear method [15]. Briefly, subjects with normal left ventricular mass (males ≤ 115; females ≤ 95, gm/m^2^) can have either concentric remodeling (normal left ventricular mass with increased relative wall thickness > 0.42) or normal geometry (relative wall thickness ≤ 0.42). Subjects with increased left ventricular mass can have either concentric (relative wall thickness > 0.42) or eccentric (relative wall thickness ≤ 0.42) hypertrophy. These measurements are based on linear method and the classification of mild, moderate, or severe abnormal values for left ventricular mass index were those proposed by the American Society of Echocardiography [15].

Flow-mediated dilation was performed as previously reported [16,17], using a Hewlett Packard ultrasound (model DR5315, USA) and measured with a high frequency ultrasound scanning probe (7 MHz).

Carotid intima media thickness (cIMT) was determined using B-mode ultrasound following the consensus statement from the American Society of Echocardiography Carotid Intima-Media Thickness Task Force [18]. A mean of two or three measurements of cIMT for both the right and left carotid arteries was used for analyses. All measurements were made in a blinded manner by the same qualified sonographer. The intra-sonographer variability in the cIMT measurements was 0.05±0.02 mm.

### Statistical analysis

Continuous variables with Gaussian distribution were compared by unpaired t test or ANOVA-Tukey test when appropriate. Non-Gaussian variables were compared by Kruskall-Wallis test. Pearson’s or Spearman’s correlation coefficients were used to evaluate the relationship between left ventricular mass with cIMT, FMD, or hormones. Categorical variables were compared by Chi-square test. A two-sided p value < 0.05 was considered statistically significant. All analyses were performed using SPSS version 21 (SPSS, Inc, Chicago, IL).

## Results

### Study population

Major characteristics of study population are shown in Table 1. Both male and female athletes had very similar exercise training programs, corresponding to two long distance running sessions every day, 15 km in the morning and 10 km in the afternoon, and intensive running training performed twice a week, corresponding to 100–1,000 meter shots, repeated many times, on Tuesday and Thursday mornings. Athletes of both genders had similar body mass index and waist circumference and these parameters were lower than those observed in healthy controls. Male and female athletes did not differ in both distance (124±25 vs. 128±29 km per week, p = 0.88, respectively, unpaired t test) and time spent in training (14±4 vs. 14±7 hours per week, p = 0.53, respectively, unpaired t test). Despite the exposure to the same training regimen, male athletes reported better mean time for 10 km than female athletes (32.4±2.1 vs. 37.6±1.6 min, p<0.0001, unpaired t test). Male athletes informed higher caloric intake, mainly due to the higher carbohydrate consumption (Table 1).

**Table 1:** Major characteristics of study population, by group.

	**Male Athletes (n = 28)**	**Male Controls (n = 24)**	**Female Athletes (n = 24)**	**Female Controls (n = 33)**	**P Value**
Age, years	30 (6)	34 (7)	33 (7)	31 (8)	0.15
Weight, kg	61 (5)	85 (15)	51 (6)	65 (14)	<0.001^a^
BMI, kg/m^2^	21 (1)	27 (4)	20 (1)	26 (5)	<0.001^b^
Glucose, mg/dL	85 (7)	91 (9)	84 (8)	87 (9)	0.008^c^
TC, mg/dL	152 (27)	188 (35)	170 (26)	181 (39)	<0.001^d^
HDL-C, mg/dL	59 (13)	47 (11)	72 (15)	61 (14)	<0.001^e^
LDL-C, mg/dL	85 (19)	117 (30)	86 (25)	105 (34)	<0.001^f^
TG, mg/dL	53 (16)	117 (63)	58 (19)	75 (27)	<0.001^g^
hsCRP, mg/L	1.2 (1.4)	2.5 (3.0)	1.6 (2.3)	3.4 (4.8)	0.056
ApoA1, mg/dL	151 (20)	133 (19)	163 (20)	149 (26)	<0.001^h^
ApoB, mg/dL	70 (14)	99 (27)	70 (17)	83 (23)	<0.001^i^
FMD, %	14.4 (10.7)	9.2 (5.2)	13.1 (12.6)	17.8 (13.8)	0.325
Systolic BP, mm Hg	111 (13)	116 (11)	109 (19)	106 (10)	0.081
Diastolic BP, mm Hg	68 (10)	74(8)	61 (19)	69 (8)	0.001^c^
Heart rate, bpm	59 (9)	72 (9)	59 (8)	76 (10)	<0.0001^i^
Diet, kcal/day	2883 (915)	2113 (707)	2298 (824)	1877 (702)	<0.0001^j^
Proteins, %	18 (7)	16 (5)	18 (6)	18 (6)	0.716
Carbohidrates, %	69 (23)	55 (9)	61 (9)	55 (9)	0.009^j^
Fats, %	28 (18)	28 (7)	21 (6)	27 (7)	0.298
Fibers, g	30 (14)	20 (9)	25 (20)	17 (9)	0.009^k^
Androstenedione	75 (25)	62 (23)	92 (42)	103 (58)	0.019^l^
11-deoxycortisol	35 (43)	32 (28)	19 (10)	19 (13)	0.255
Testosterone	539 (192)	359 (182)	22 (14)	25 (13)	<0.000^m^
17-α Hydroxyprogesterone	97 (59)	51 (24)	96 (75)	55 (55)	0.016^j^
Corticosterone	333 (275)	256 (205)	222 (149)	189 (147)	0.152

Values are mean (SD). TC–total cholesterol; TG–triglycerides; hsCRP–highly-sensitive C-reactive protein; FMD–flow-mediated dilation; BP–blood pressure; Sex hormones are ng/dL.

Comparisons were made by ANOVA-Tukey.

^a^Male controls > other groups; female controls > female athletes; male athletes > female athletes

^b^Male controls > other groups; female controls > male and female athletes

^c^Male controls > female athletes

^d^Male athletes < female athletes and male controls

^e^Male controls < other groups; female athletes > other groups

^f^Male and female controls > male and female athletes

^g^Male controls > other groups

^h^Male controls < other groups

^i^Male controls > male and female athletes

^j^Male athletes > male and female controls

^k^Male athletes > female controls

^l^Female controls > male controls

^m^Male athletes > other groups; male controls > female athletes and female controls.

### Laboratory parameters

Lower levels of total cholesterol, LDL-cholesterol, and triglycerides were found in athletes of both genders, with higher HDL-cholesterol levels in female athletes. Male controls had lower levels of apolipoprotein A1 and higher levels of apolipoprotein B than other groups (Table 1). Higher serum levels of androstenedione were observed in female controls, whereas testosterone was higher in male athletes than other groups. In addition, levels of 17α-hydroxyprogesterone were higher in male and female athletes compared with male and female controls. No differences were observed for deoxycortisol or corticosterone (Table 1). Testosterone levels and LV mass were positively correlated (r = 0.501, p<0.0001, Spearman). Female athletes and controls did not report disturbances on their menstrual cycles.

### Ultrasound measurements

**Fig 1:**
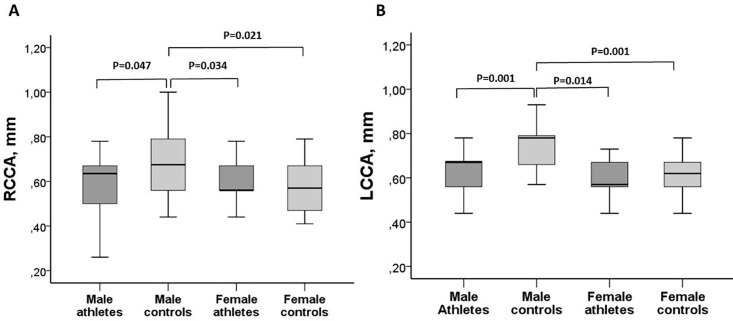
Carotid intima-media thickness, by study groups. A- Right common carotid artery (RCCA). B- Left common carotid artery (LCCA). Comparisons were made by ANOVA-Tukey.

Male controls had higher carotid intima-media thickness (cIMT) of the right and left common carotid arteries when compared with other groups. However, while cIMT was lower in male athletes than in male controls, for left and right common carotid arteries, no differences were found for any of these parameters between female athletes and female controls (Fig 1).

**Fig 2:**
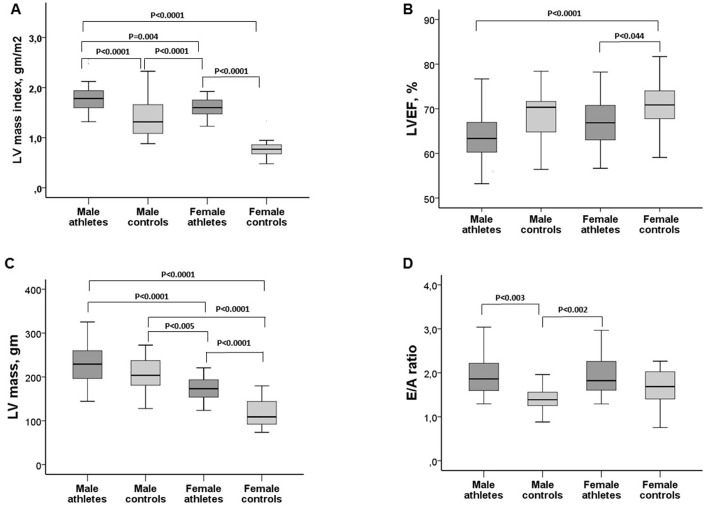
Echocardiographic characteristics of the study population, by group. A-Left ventricular mass index (LV mass index). B-Left ventricular Ejection Fraction (LVEF). C-Left ventricular mass (LV mass). D-Early (E) to late (A) ventricular filling velocities ratio (E/A ratio). Comparisons were made by ANOVA-Tukey.

Higher left ventricular (LV) mass and LV mass index were observed in male athletes when compared with other groups (Table 2). The degree of left ventricular mass classified as mildly, moderately or severely abnormal was observed in 26%, 35%, and 30%, respectively, of female athletes, and in 39%, 14%, and 21%, respectively, of male athletes. In controls, 18% of males had mild increase in ventricular mass, with normal ventricular mass observed in females. Male and female athletes presented lower LV ejection fraction when compared with female controls. However, the E/A ratio was higher among athletes of both genders than male controls (Fig 2). Other echocardiographic parameters and differences between groups are shown in Table 2.

**Table 2:** Echocardiographic parameters, by group.

	**Male Athletes (n = 28)**	**Male Controls (n = 19)**	**Female Athletes (n = 23)**	**Female Controls (n = 23)**	**P Value**
LA diameter, mm	33 (3)	35 (5)	33 (4)	33 (4)	0.32
LA volume, mm^3^	206 (24)	192 (31)	185 (17)	170 (31)	<0.04^a^
RV, mm	23 (5)	19 (6)	21 (5)	19 (4)	0.052
LV systolic, mm	34 (3)	29 (5)	30 (3)	29 (4)	<0.001^a^
LV diastolic, mm	52 (3)	48 (5)	49 (4)	48 (6)	<0.004^b^
Septum wall, mm	11 (1)	9 (1)	10 (1)	9 (2)	<0.0001^c^
Posterior wall, mm	9 (1)	8 (1)	8 (1)	8 (1)	<0.002^a^
IVRT, msec	105 (18)	90 (14)	98 (16)	87 (13)	<0.0001^c^
LV mass, g	229 (43)	163 (58)	173 (28)	162 (65)	<0.0001^d^
Mass index, g/m^2^	132 (24)	86 (24)	116 (14)	87 (26)	<0.0001^e^
Aorta, mm	25 (3)	27 (4)	22 (3)	25 (4)	<0.0001^f^
LVEF, %,	64 (6)	69 (8)	67 **(**6)	70 (6)	0.002^g^
E/A ratio,	2.0 (0.5)	1.4 (0.4)	2.0 (0.6)	1.7 (0.5)	0.002^h^
E’/A’ ratio,	1.7 (0.4)	1.5 (0.6)	2.0 (0.6)	1.7 (0.5)	0.035^i^
E/e’ ratio,	6.4 (1.0)	6.3 (1.1)	6.8 (1.4)	6.4 (1.5)	0.594

Values are mean and standard deviation (SD). LA–left atrium; RV–right ventricule; LV–left ventricular; IVRT–Isovolumic relaxation time; LVEF–Left ventricular ejection fraction; E/A—the ratio of the early (E) to late (A) ventricular filling velocities; E’/A’- early (E′) and late (A′) peak velocities of septal and lateral mitral annulus; E/e’- early mitral inflow velocity (E) and mitral anular early diastolic velocity (e’) ratio.

Comparisons were made by ANOVA-Tukey.

^a^Male athletes > female athletes and female controls

^b^Male controls > other groups

^c^Male athletes > male and female controls

^d^Female controls < other groups; female athletes < male athletes and male controls

^e^Male athletes > other groups; female athletes > male controls and female controls

^f^Female athletes < male athletes and male controls

^g^Female controls > male athletes and female athletes

^h^Male and female athletes > male controls

^i^Male controls < other groups.

Flow-mediated dilation (FMD) did not differ between groups (Table 1). However, an inverse relationship between FMD and cIMT was found when male and female controls were grouped (r = -0.49, p<0.0001, Spearman), but not in athletes of both genders (r = -0.08, p = 0.564, Spearman). Systolic blood pressure levels were similar between groups, but diastolic blood pressure levels were lower in female athletes than male controls (Table 1). Heart rate was lower in male and female athletes than in male controls (Table 1).

## Discussion

Our study reports the effects of long-term training of professional runners on cardiovascular system, and describes some differences between genders, in spite of same training regimen.

We found a clear impact of exercise on LV mass in comparison with controls, with the majority of runners exhibiting LV mass classified as mildly or moderately abnormal. Despite some increase in cardiac cavities and reduced LV ejection fraction, mainly observed among male athletes, several other parameters including those related to diastolic function, suggest a physiological remodeling, based on the higher E/A and E’/A’ ratios.

The relevance of left ventricle hypertrophy (LVH) found in many athletes has been reviewed, particularly regarding the differentiation between the athlete’s heart from primary cardiomyopathy. In addition, severe forms of LVH have been rarely reported in athletes [19,20]. More recently, new echocardiographic parameters have been proposed for subjects in the gray zone (defined by wall thickness between 13–15 mm) aiming to identify subjects with LVH related to exercise and those with primary cardiomyopathy [21]. The authors found lower LV cavity (< 54 mm) in primary cardiomyopathy as the main finding in comparison with LVH present in athletes [21]. However, this parameter should be applied only for those subjects with severe wall thickness (13–15 mm). In our study the prevalence of LV ≥ 54 mm was similar between athletes and controls, but only a minority of subjects had wall thickness of 13 mm or greater. The higher E/A ratio, reflecting the ratio of the early (E) to late (A) ventricular filling velocities, observed in athletes, suggests a healthy adaptation of the heart, even with higher ventricular mass. These results contrast with lower E/A ratio reported among amateur runners, suggesting left ventricular dysfunction during ultradistance trail running [22].

Some differences between male and female athletes were observed for carotid IMT. We found lower carotid IMT only among male athletes when compared with their controls. Carotid IMT has been considered a marker of subclinical atherosclerosis [23] and the lack of benefit in the athlete women suggests that intense exercise regimen may not protect them against the development of atherosclerosis. However, the cIMT values obtained for female athletes were in the normal range reported for the general population, according to gender and age [24,25]. In our study we measured right and left common carotid arteries intima-media thickness as a validated marker for prediction of coronary risk, based on the large cohort of The Atherosclerosis Risk in Communities (ARIC) study [26].

Interestingly, the increased levels of testosterone in male athletes possibly contributed to better exercise performance. It has been reported that the use of synthetic androgenic-anabolic steroids may improve exercise performance [27], but it is associated with persistent long-term disturbances on the endogenous production of testosterone and gonadotropins with deleterious effects on cardiovascular system, including vasospasm, thrombosis and dyslipidemias [27–29]. In our study, the increased levels of testosterone found among male runners in comparison to male controls seems related to the exercise training and not to steroid supplementation [30,31].

We found very good metabolic profile in athletes of both genders, based on serum levels obtained for lipids, lipoproteins, glucose, hsCRP and anthropometric data. The daily energy intake and components of the diet were also comparable among male and female athletes.

## Conclusion

The exposure to intense, long-term exercise regimen in professional runners of both genders was associated, in general, with mild to moderate left ventricular hypertrophy, without impairment in diastolic function. In spite of optimal metabolic and anthropometric parameters, lower diastolic blood pressure and heart rate in athletes of both sexes, lower degree of subclinical atherosclerosis was observed only in male athletes. Differences in steroid hormones may contribute in part to these findings.

### Practical implications

- High-intensity training in professional runners has different impact on subclinical atherosclerosis and ventricular mass;
- Despite similar and optimal metabolic profile in professional runners of both genders, lower carotid intima-media thickness was found only in male athletes;
- The carotid intima-media thickness in female athletes was similar to female controls;
- Increased ventricular mass predominantly of mild to moderate degree was observed among professional runners of both genders;
- Differences in sex hormones may explain in part our findings.

## Supporting Information

S1 Fig intima-media thickness, by study groups. A- Right common carotid artery (RCCA). B- Left common carotid artery (LCCA).
(SAV)

S2 Fig Echocardiographic characteristics of the study population, by group. A-Left ventricular mass index (LV mass index). B-Left ventricular Ejection Fraction (LVEF). C-Left ventricular mass (LV mass). D-Early (E) to late (A) ventricular filling velocities ratio (E/A ratio).
(SAV)

S1 Table Major characteristics of study population, by group. (SAV)

S2 Table Echocardiographic parameters, by group. (SAV)
